# Anesthetic Management in a Gravida with Type IV Osteogenesis Imperfecta

**DOI:** 10.1155/2016/7429251

**Published:** 2016-06-28

**Authors:** Elizabeth Vue, Juan Davila, Tracey Straker

**Affiliations:** Montefiore Medical Center, 111 East 210th Street, The Bronx, NY 10467, USA

## Abstract

Osteogenesis imperfecta (OI) is an inherited disorder of the connective tissues caused by abnormalities in collagen formation. OI may present many challenges to the anesthesiologist. A literature review reveals a wide range of implications, from basic positioning to management of the difficult airway. We present the anesthetic management of a 25-year-old gravid woman with OI, fetal demise, and possible uterine rupture, admitted for an exploratory laparotomy.

## 1. Introduction

Osteogenesis imperfecta (OI) is a genetic connective tissue disorder, commonly known as brittle-bone disease, with different phenotypic presentations due to quantitatively insufficient or qualitatively abnormal type 1 collagen [1–3]. It commonly manifests with multiple bone fractures and may be accompanied by a reduced life span. Inherited autosomal dominant gene mutations in the alpha chain that comprise type 1 collagen account for approximately 90% of OI cases [3, 4].

Subtypes of OI are characterized based on genetic, radiographic, and clinical findings [4–6]. Symptoms, ranging from mild to severe, may present at any age. Common clinical manifestations of OI include fractures, with sometimes absent to minimal trauma, blue sclera, short stature, scoliosis, limb deformities, joint laxity, and platelet dysfunction [4–11].

We present the case and discuss the anesthetic challenges in the management of a gravid patient with type IV OI presenting with possible uterine rupture. The challenges encountered include complex airway management, respiratory compromise secondary to skeletal deformity, dwarfism, and potential fractures from positioning.

## 2. Case Report

A 25-year-old wheelchair bound multiparous woman at 18 weeks of gestation with a history of OI, scoliosis, and dwarfism presented with complaints of abdominal pain, nausea, and vomiting. The patient denied any history of cardiac disease. She did give a history of not being able to be ventilated or intubated during her last caesarean section, culminating in an emergent tracheostomy that was later removed. Based on her medical history and physical exam, she appeared to have a moderate form of OI.

On admission to the ER, no fetal heart tones were noted. CT findings revealed expanding hemoperitoneum from a possible uterine rupture. Initial assessment pointed to hypovolemic shock. On physical exam, the patient was 41 inches tall (3′5′′), weighing 37 kg, with flexed upper extremities and short, bowed legs. Airway examination revealed a short neck with limited neck extension, two-fingerbreadth thyromental distance, small mouth opening, Mallampati class 3, poor dentition and a tracheostomy scar. Her abdomen was grossly gravid with an umbilical hernia and was tender to light palpation. The patient was alert and oriented, in visible pain and distress, while recumbent in the fetal position. Preoperative vitals and pertinent labs were as follows: temperature 97.9°, HR 110 BP 70–100/30–40, RR mid-20 s, and SpO_2_ 99-100% on room air.

WBC 20.9 k/*μ*L, H/H 6.1/19.1, platelets 243 k/*μ*L, sodium 139 mEq/L, potassium 3.4 mEq/L, chloride 109 mEq/L, bicarbonate 10 mEq/L, urea nitrogen 13 mEq/L, creatinine 0.51 mg/dL, glucose 366 mg/dL, calcium 7.1 mg/dL, and lactic acid 5.7 mmol/L were found. The venous blood gas revealed pH 7.1, partial pressure of carbon dioxide 41.3 mmHg, partial pressure of oxygen 38.9 mmHg, base excess −15.3 mmol/L, hemoglobin 4.6 g/dL, and glucose 214 mg/dL.

A right femoral triple lumen catheter was emergently placed in the ER. The patient's hemodynamic status improved with normal saline resuscitation and packed red blood cells (PRBCs).

The patient was transported to the OR for an emergent exploratory laparotomy. On patient sign-in with the anesthesia team, OR nursing staff, and surgical team present, the patient confirmed the procedure written on the consent form, including a possible hysterectomy if it was life-threatening. She emphasized her desire to maintain fertility for future pregnancies if possible.

The anesthetic plan, potential complications, and the Do Not Resuscitate (DNR) status were subsequently addressed to the patient and the anesthesia team. Both the OR nursing staff and surgical staff were present for the discussion. The patient indicated her understanding and gave verbal consent for full resuscitative measures in the perioperative period. American Society for Anesthesiologist (ASA) monitors were placed and a right radial arterial line was inserted in sterile fashion in place of the noninvasive blood pressure cuff to avoid possible bony trauma. An arterial blood gas was immediately obtained.

Fiberoptic intubation was attempted to avoid possible complications from direct laryngoscopy, anticipated difficult airway, and use of succinylcholine. No sedation was given secondary to possible risk of aspiration, hemodynamic instability, and desaturation in the setting of a difficult airway. The airway was topicalized with aerosolized 4% lidocaine and a negative gag reflex was achieved. The initial fiberoptic intubation was attempted unsuccessfully via an Ovassapian airway. There was limited mouth opening, and the mouth was small as well. These features made placement of the Ovassapian airway difficult. The patient had a persistent gag reflex despite additional topicalization through an epidural catheter threaded in the bronchoscope port. A second fiberoptic attempt was made through a slit 22 F nasopharyngeal trumpet placed in the left nostril. The nares were tight and edematous. These features made it difficult to pass the bronchoscope down the correct passage to the nasopharyngeal space. Despite the distorted anatomy, the nasal fiberoptic approach proved successful. A gradual inhalational induction and maintenance with sevoflurane was instituted to avoid severe hypotension.

The patient remained hemodynamically stable throughout the intubation process. A 14 G right external jugular intravenous line was placed. The patient remained normothermic via an upper body Bair Hugger*™* and a fluid warmer. The baseline intraoperative arterial blood gas (ABG) revealed pH 7.49, partial pressure of carbon dioxide 19 mmHg, partial pressure of oxygen 144 mmHg, base excess −8.1 mmol/L, lactic acid 4.2, hemoglobin 6.2 g/dL, and glucose 128 mg/dL. Upon initial surgical approach, 2 L of blood was evacuated from the abdomen. Hemostasis was obtained quickly. Aggressive resuscitation instituted at the time included 3 L of crystalloids and 4 U of PRBCs. Further exploration of the abdominal contents confirmed a ruptured uterus with an extra-uterine fetal demise.

The option of hysterectomy was discussed by the obstetricians and gynecologic-oncologists due to the increased risk of morbidity and mortality for this patient. The surgeons were primarily concerned about possible uterine rupture and postpartum hemorrhage in subsequent pregnancies. Secondary concerns regarding this patient included poor prenatal care and failure to obtain anesthesiology consultation prior to admission for delivery. The anesthetic consultation was felt necessary for team-based clinical management planning due to the high-risk nature of this patient's condition. In response to the considerations for hysterectomy while in the operating room, the anesthesiologist advocated for the patient's wishes, reemphasizing her desire to maintain fertility, if possible. The uterus was repaired and conserved. The patient was transported to SICU and was successfully extubated on postoperative day #1 without complications.

## 3. Discussion

Osteogenesis imperfecta is one of the most common skeletal dysplasias, estimated around 6-7 per 100,000 births [4, 6]. It is an extremely heterogeneous group of heritable connective tissue disorders that was first classified into four major types by Sillence et al. in 1979 [7]. More recent studies have currently identified 17 causative genes, including autosomal recessive genes [4, 6]. As a result, the nomenclature and classification have evolved substantially. In 2009, the International Nomenclature Group for Constitutional Disorders of the Skeleton (INCDS) standardized the classification of OI [4, 6] based on the severity and clinical features of the disorder (see Table 1).

Patients with moderate-severe OI are susceptible to bone fractures, bruising, and dislocation from minimal to no trauma [5, 8]. One should exercise care during transportation, placement on the operation table, and positioning. For some patients, supine position with fully extended arms abducted less than 90 degrees may cause perioperative morbidity. Pressure points should be supported and padded. The placement of tourniquets [8] for the insertion of peripheral intravenous (IV) catheters must be approached with caution. Arterial cannulation for blood pressure measurement may be preferred to avoid repeated trauma from a blood pressure cuff [12]. Bleeding and bruising tendencies in these patients are well documented [2, 11–14].

Excessive bone fragility can be challenging for the anesthesiologist. Abnormal skeletal growth causing anatomical distortion of the airway may impede tracheal intubation [8, 9, 11]. Neck and mandibular fractures may occur during laryngoscopy. Upward translocation of the cervical spine (basilar invagination) [4, 6] may disrupt vascular and cerebral spinal fluid flow and result in hindbrain herniation. Presence of dentinogenesis imperfecta (DI) in patients with OI increases the risk of tooth dislodgement during oral instrumentation [6, 8, 9, 11, 12]. In appropriate clinical scenarios, supraglottic airways [8, 9, 11, 12] have been safely placed to prevent the complications that might arise from tracheal intubation. Regional anesthesia can be safe and effective [10–12]; however; a thorough assessment of the airway, severity of spine deformity, prior back surgery, and platelet function should be done.

Avoidance of succinylcholine should be considered due to the potential for fasciculation-induced fractures [8, 11, 12] and malignant hyperthermia (MH). Studies have looked into the association of OI and hyperthermia with or without MH susceptibility [15, 16]. A review of cases involving MH and caffeine halothane contracture test (CHCT) showed results in the context of coexisting diseases and syndromes [15]. It was concluded that there is weak evidence for the association of OI to MH, but a positive association to intraoperative hyperthermia responsive to standard cooling methods. Within these case series, most patients with OI were found to have normal CHCT, and no reports of MH were confirmed in OI patients with positive CHCT. In contrast, a retrospective study showed no significance in intraoperative hyperthermia or end-tidal CO_2_ levels between patients with OI and those undergoing general anesthesia [16], including use of sevoflurane. Ogawa et al. [17] advocates using total intravenous infusion (TIVA) to avoid body temperature elevation and MH; however there may be a risk of propofol infusion syndrome as reported in one case after short-term propofol infusion for anesthesia [17, 18].

Patients with OI may not tolerate general anesthesia well. Spinal and chest wall deformities predispose patients with OI to pulmonary disease, ventilation-perfusion mismatch, and rapid desaturation [19]. Pectus carinatum and kyphoscoliosis limit thoracic movement and lung expansion resulting in restrictive pulmonary disease. These derangements include decreased vital capacity, decreased functional residual capacity, and decreased chest wall compliance. Reduced functional residual capacity of pregnancy adds to pulmonary compromise [20]. Hemodynamic changes should be anticipated from aortocaval compression from the gravid uterus combined with the vasodilatory effects of general anesthesia. Furthermore, these patients may have cardiac disease including valvular dysfunction.

Platelet dysfunction is a clinical concern commonly encountered [11, 14]. Preoperative platelet transfusion should be considered. Studies have shown increased capillary fragility, decreased platelet retention, decreased factor VIII production, and decreased collagen-induced platelet aggregation. The underlying collagen abnormality [14] can result in delicate tissues and small blood vessels that are unable to adequately constrict. Pregnant women are prone to uterine atony and may result in excessive postpartum hemorrhage or disseminated intravascular coagulopathy [10, 11, 20] (see Table 2).

Pregnant women with OI who have skeletal deformity and short stature should be monitored in high-risk prenatal care centers for both maternal and fetal safety [20]. Breech presentation of the fetus is common in women with OI. In addition, pregnant women with OI usually do not tolerate the increasing size of a gravid uterus due to short stature and usually require early caesarean section [6, 12, 20]. Spontaneous uterine rupture has been described in women with OI [2, 3]. Increased risk of vaginal lacerations and uterine rupture have also been described in women attempting vaginal delivery and are often treated as a trial of labor with a scarred uterus [12]. Studies have shown that women with OI have a decreased amount of collagen type I in the myometrium [2]. This is thought to be the underlying cause of spontaneous uterine ruptures. However, women with OI have also successfully delivered without complications [3]. Literature reports both successful vaginal and cesarean births in these patients and the optimal mode of delivery should be decided on an individual basis [20].

### 3.1. Ethical Concerns

The ASA Guidelines and American College of Surgeons recommend that prior to procedures requiring anesthetic care, any existing directives limiting the use of resuscitative methods should be reviewed with the patient or designated surrogate when possible [21–23]. Automatic suspension of DNR orders in the OR is inappropriate without informed consent. There is agreement that opportunities for a careful, informed discussion about the potential resuscitative measures and surgical risks should be discussed in order to provide the treatment that best supports the patient's vision of care and the acute clinical situation. If possible, all physicians in the healthcare team directly involved in the care of the patient during the procedure should be present and included [21]. As a result, these directives should be clarified or modified based on the preference of the patient and clearly documented in the medical record.

Fertility is usually preserved [11, 20] in patients with mild to moderate OI. Though there are increased risks for uterine rupture and postpartum hemorrhage, patients with OI have also successfully delivered without complications. There may be circumstances when adhering to the patient's wishes is likely to result in harm and cause surgical teams and anesthesiologists to hesitate in their actions [23]. However, patient autonomy is essential to ethical decision-making. It is the responsibility of the provider to address and advocate for the patient's right. Not adhering to the patient's desire may cause harm to both the patient and family, as well as to the provider. Healthcare workers are often impacted by medical errors in the workplace and suffer as “second victims” [24, 25]. Studies have described damage to the provider's confidence and self-esteem, exhibiting posttraumatic stress symptoms and requiring support. Second victim is a common problem for healthcare organizations, and trainees are particularly more vulnerable [25]. This example reemphasizes the importance for an open discussion addressing the patient's goal of care and the surgical and anesthetic concerns prior to a procedure. If the provider finds the medical decisions for patients to be irreconcilable with his own moral views, then the provider should withdraw in a nonjudgmental fashion, providing an alternative plan for care in a timely fashion [21].

## 4. Conclusion

Patients with OI pose significant challenges for the anesthesiologist. In the past, many patients with moderate to severe OI died by the end of the second decade, mainly due to complications of skeletal chest wall deformity and cardiorespiratory failure [19]. With the current therapeutic options available, the majority of these patients will survive into adult life. These patients may present with a wide range of obstacles that should be considered and managed appropriately. The ability to identify the type of OI a patient has and to consider associated clinical conditions will help determine the choice of perioperative anesthetic management. An anesthesiology consultation prior to an elective surgery and in early pregnancy is recommended. In the setting of an emergent procedure, it is prudent to try to attain a thorough preoperative assessment and devise a preinduction anesthetic plan as this may improve outcomes in these patients.

After a thorough literature research using PubMed, Google Scholar, and Ovid Medline, and Cochrane, our recommendations for anesthetic management of these cases include the following:

1. Preoperative:
  - Discontinue bisphosphonate infusions from the start of pregnancy [20] if the patient is on the regimen. Exposure could cause skeletal abnormalities and congenital malformations.
  - Obtain detailed medical history to determine the type and severity of the patient's disease [4, 6].
  - Obtain echocardiography to evaluate cardiac anatomy and function [4], if indicated.
  - Accurately assess the airway to determine difficulty of intubation [8, 9, 11].
  - Devise the anesthetic plan and alternative plans.
  - Confirm a type and screen [11, 14].
2. Intraoperative:
  - Transport and position patient with care [5, 8].
  - Consider arterial cannulation in place of blood pressure cuff to avoid bone fractures and bruising [11–14].
  - Avoid succinylcholine use [8, 11, 12] if feasible for patient clinical management.
  - Be vigilant of hemodynamic and ventilation changes [11, 14, 19, 20].
  - Be cognizant of the risk for hyperthermia and malignant hyperthermia [15, 16].
  - Monitor for excess bleeding [11, 14].
3. Postoperative:
  - Ensure adequate oxygenation and ventilation.
  - Monitor for postoperative hemorrhage.
  - Pain control: patients with OI may have chronic bone pain that is not related to surgical site.
  - Consider resuming cyclic intravenous pamidronate therapy postoperatively [13] and for a minimum of 2 years [26]. Most studies are seen in pediatrics.
  - Bisphosphonate use as analgesics have been shown to be beneficial in CRPS, osteoporosis, Paget's disease of the bone, multiple myeloma, metastatic bone disease, and vertebral compression fractures [27, 28].

## Figures and Tables

**Table 1:** Classification of osteogenesis imperfecta and related anesthetic concerns [4, 6].

Types	OI syndromic names	Gene	Inheritance	Postnatal clinical characteristics	Anesthetic concerns
Type I	Nondeforming OI with blue sclera	COL1A1 COL1A2	AD AD	Rarely congenital fractures; low bone mass; deformity of spine or long bones is uncommon; higher frequency of long bone fractures in presence of dentinogenesis imperfecta (DI); near normal growth velocity and height; ambulant; blue-gray sclera; susceptible to conductive hearing loss; absence of chronic bone pain or minimal pain controlled by simple analgesics	Bone fractures during extremity manipulation (e.g., positioning, PIV placements with tourniquet), dental damage during oropharyngeal instrumentation, difficulty of hearing, hyperthermia or malignant hyperthermia, platelet dysfunction, capillary fragility

Type II	Perinatally lethal OI	COL1A1 COL1A2 CRTAP LEPRE1 PPIB	AD AD AR AR AR	Ribs with continuous or discontinuous fracture; crumpled (accordion-like) long bones and multiple fractures; thighs abducted and in external rotation; all vertebrae hypoplastic/crushed; clinical indicators of severe chronic pain; small thorax; respiratory distress leading to perinatal death	Most prenatally diagnosed pregnancies are terminated. Rarely do these patients survive to adulthood. Pain relief is valuable

Type III	Progressively deforming	COL1A1 COL1A2 BMP1 CRTAP FKBP10 LEPRE1 PLOD2 PPIB SERPINF1 SERPINH1 TMEM38B WNT1 CREB3L1	AD AD AR AR AR AR AR AR AR AR AR AR AR	Usually near term; newborn or infant presentation with bone fragility and multiple fractures; platyspondyly vertebrae at birth; thin ribs with discontinuous beading/fractures; marked short stature; progressive kyphoscoliosis and bowing of legs; generalized osteoporosis/osteopenia; increased prevalence of basilar impression; possibly having blue sclera at birth; DI is variable; hearing loss is more frequent in adults; possibly having cardiovascular complications such as valvular dysfunction or aortic root dilation	Bone fractures during extremity manipulation, posterior fossa compression syndromes due to basilar impression from cervical manipulation, pulmonary insufficiency or hypertension, cardiopulmonary failure, hyperthermia or malignant hyperthermia, platelet dysfunction, capillary fragility, postoperative pain control

Type IV	Common variable OI with normal sclera	COL1A1 COL1A2 WNT1 CRTAP PPIB SP7 PLS3	AD AD AD AR AR AR XL	Variable severity; recurrent fractures; vertebral compression fractures; osteoporosis; variable degrees of deformity of long bones and spine (thoracolumbar kyphoscoliosis); bowing of long bones; short stature, possibly being wheelchair bound; normal sclera; DI; increased prevalence of basilar impression (5 times higher relative risk in those with DI); hearing impairment is not often encountered; possibly having chronic bone pain; possibly having cardiovascular complications such as valvular dysfunction or aortic root dilation	Bone fracture or dislocation, dental damage, posterior fossa compression syndromes, pulmonary insufficiency or hypertension, cardiorespiratory failure, hyperthermia or malignant hyperthermia, platelet dysfunction, postoperative pain control

Type V	OI with calcification in interosseous membranes	IFITM5	AD	No congenital fractures; distinguished by calcification of interosseous membrane in forearms; increased risk of developing hyperplastic callus; restriction of pronation and supination of forearms; radial head dislocations; bowing of long bones in some patients; vertebral compression fractures; no DI presence; white sclera	Bone fractures and dislocations during extremity manipulation, indomethacin recommended to avert callus progression, hyperthermia or malignant hyperthermia, platelet dysfunction, capillary fragility

**Table 2:** Osteogenesis imperfecta challenges in the perioperative setting.

Perioperative phase	OI specific anesthetic challenge
Preoperative	Hypovolemic shock Blood loss Anticipation of difficulty of intubation NPO status Gravida status

Intraoperative	Positioning Hemodynamic monitoring: noninvasive versus invasive PIV placement Airway control: tracheal intubation technique Dental and oral trauma avoidance General anesthetic agent: inhalational versus TIVA Ventilation and oxygenation management Hemodynamic management Hemostasis Extubation

Postoperative	Ventilation and oxygenation management Pain control
